# Low-Dose Arsenic: In Search of a Risk Threshold

**DOI:** 10.1289/ehp.122-A130

**Published:** 2014-05-01

**Authors:** Charles W. Schmidt

**Affiliations:** **Charles W. Schmidt**, MS, an award-winning science writer from Portland, ME, has written for *Discover Magazine*, *Science*, and *Nature Medicine*.

Scientists long ago linked high levels of arsenic in groundwater to cancer and other environmental illnesses, particularly in Taiwan, Bangladesh, and South America, where the contamination can often reach extraordinarily high levels of 1,000 ppb or more. Now concerns are shifting to the health effects of much lower doses such as those that many Americans live with every day.

**Figure:**
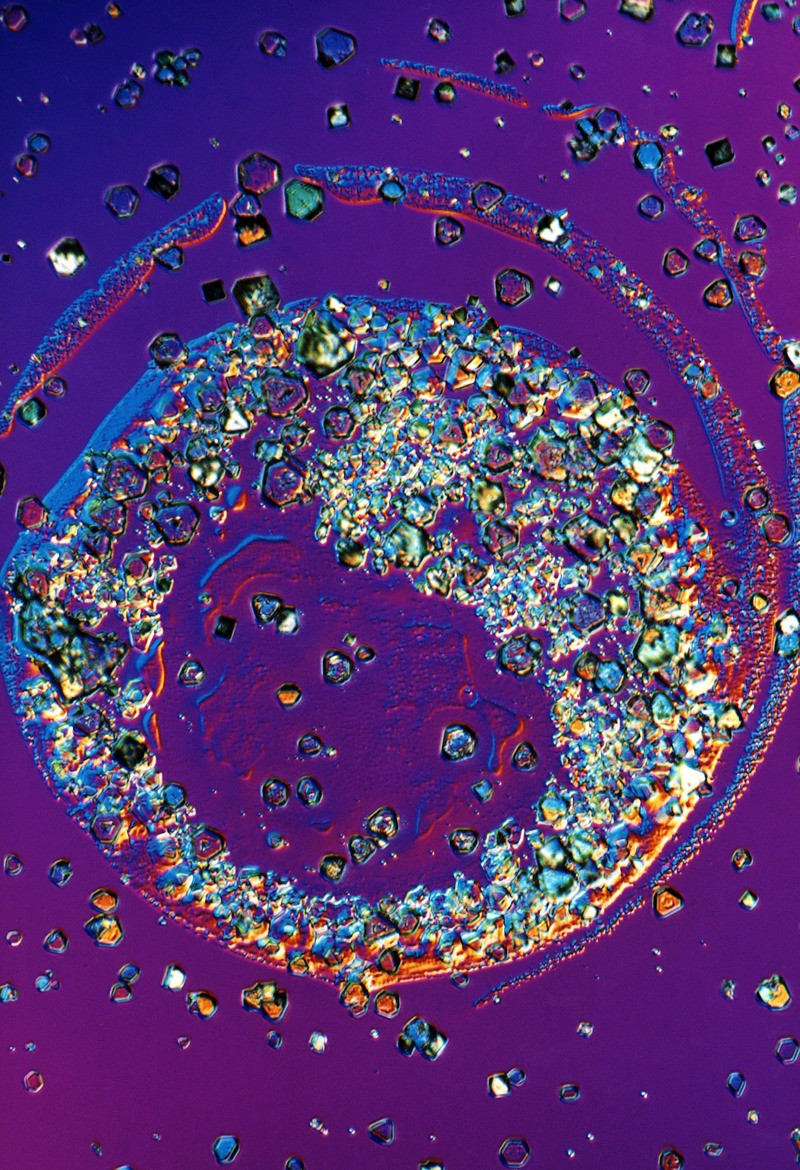
Naturally occurring crystals of inorganic arsenic crystals, magnification unknown. © Manfred Kage/Science Source

Margaret Karagas, who directs the Children’s Environmental Health and Disease Prevention Research Center at Dartmouth College, says researchers increasingly believe that arsenic risks are more widespread than previously recognized, particularly during vulnerable periods such as pregnancy and childhood. Protecting against low-level exposure is challenging, however, given that arsenic is a natural element in the Earth’s crust and ubiquitous throughout the environment.

Moreover, the evidence for low-dose effects is controversial. One view holds that arsenic has a dose threshold below which exposures aren’t harmful. But controversial studies in the peer-reviewed literature increasingly suggest this threshold may not exist, so that any exposure—no matter how small—could boost risks for diabetes, heart disease, immunological problems, and cancer.^1^^,^^2^^,^^3^^,^^4^^,^^5^^,^^6^

The disagreement is a problem for regulators who face mounting pressure to set or reduce standards for arsenic. The U.S. Environmental Protection Agency (EPA) is grappling with a revised estimate of arsenic carcinogenicity that, if enacted, would result in unattainable clean-up standards, according to Susan Griffin, a senior toxicologist with the EPA’s Region 8 office in Denver, Colorado. The U.S. Food and Drug Administration (FDA) is also under pressure to regulate arsenic in foods, especially rice, which readily absorbs the metal as it grows, making it a top source of dietary exposure.

The focus on rice comes on the heels of a new “action level” of 10 ppb for arsenic in apple juice that was proposed by the FDA in July 2013.^7^ This new value, which tightens the agency’s previous “level of concern” of 23 ppb (and which has yet to be formally adopted), was motivated in part by mounting publicity over low doses of arsenic in the diet, including media-directed efforts by the public-interest group Consumers Union (CU) to raise awareness on the issue. Growing public scrutiny has put a spotlight on the complex question of how very low arsenic exposures may affect human health.

## A Historical View

That arsenic can be lethal has been known since antiquity. But lethal doses of arsenic are difficult to quantify, and they depend on solubility, valence states, and other factors. The Agency for Toxic Substances and Disease Registry suggests that the minimal lethal exposure in humans ranges from 1 to 3 ppm, with death resulting from cardiovascular collapse and hypovolemic shock.^8^

Researchers didn’t perceive arsenic as an environmental health threat until studies in Taiwan, and later in Chile, linked levels in groundwater with skin cancers such as squamous cell carcinoma (which is rarely fatal) and a condition called black foot disease (which affects blood vessels, leading to gangrene). Villagers were exposed to the arsenic beginning in the early twentieth century after artesian wells were drilled throughout southwestern Taiwan to avoid saltwater intrusion from shallower wells.^9^ The U.S. Public Health Service aimed to protect against the arsenic-related skin problems seen in Taiwan when it set a 50-ppb standard for arsenic in drinking water in 1942, which was then adopted by the EPA in 1975.^10^

The levels deemed “low” in early environmental health research on arsenic were much higher than what’s considered low today. Studies from Taiwan up to the 1980s described groundwater levels of up to 300 ppb as low, of up to 600 ppb as moderate, and values beyond that as high.^10^^,^^11^ These delineations were based on a view that consuming arsenic in groundwater, while harmful, wasn’t fatal in the long run.

Two pivotal studies led researchers to reconsider that point. Chien-Jen Chen, who was then a teaching assistant at the National Taiwan University College of Medicine, and colleagues showed that arsenic could, in fact, boost risks for fatal malignancies at groundwater concentrations far less than 600 ppb. Published in 1985, the first study reported statistically significant associations between chronic exposure to artesian well water in Southwestern Taiwan and elevated mortality from cancers of the lung, bladder, and other internal organs.^12^^,^^13^ And in their follow-up study, Chen and colleagues reported that this relationship was dose-dependent—i.e., that cancer rates grew with higher arsenic exposure—and that mortality rates were especially high in areas where blackfoot disease also was endemic.^11^

Joseph Graziano, a professor of environmental health sciences and pharmacology at Columbia University, says Chen’s data had far-reaching consequences that scientists are still grappling with today. Without evidence to the contrary, the EPA defaulted to what is still a standard regulatory assumption: namely that any exposure to a carcinogen, no matter how small, increases cancer risk to some degree. Therefore, the National Research Council (NRC) now describes arsenic levels beyond 150 ppb as high, between 150 ppb and 50 ppb as moderate, and below 50 ppb as low.^14^

But linear assumptions drive considerable risk even at low exposures. Extrapolating from high-dose human data, the NRC predicted in 1999 that the 50-ppb water standard could induce cancer in as many as 1 in 100 people.^15^

By that time, the EPA had already been engaged in technical review on arsenic for years. The agency ultimately evaluated more than 300 studies and drew on expert opinions from the NRC, the National Drinking Water Council, and its own Science Advisory Board (SAB) before it finally dropped the standard from 50 to 10 ppb in 2001^16^—a level the NRC estimated might lead to a cancer risk of approximately 1 in 300 for people exposed over a lifetime.^17^ According to Craig Steinmaus, an associate adjunct professor in epidemiology at the University of California, Berkeley, School of Public Health, the EPA by necessity had to factor cost and technical feasibility as well as health into the 10-ppb drinking water standard.

## Debating the Standard

The EPA’s risk assumptions on arsenic were criticized by researchers who felt it was inappropriate to extrapolate low-dose effects from the high-dose Taiwanese studies. Samuel Cohen, a professor of pathology and microbiology at the University of Nebraska Medical Center, has long maintained that arsenic has a dose threshold below which exposures are not harmful. According to Cohen’s own research with rodents (in addition to *in vitro* and *in vivo* studies by other researchers), arsenic is carcinogenic only at doses high enough to induce cytotoxicity followed by regenerative cell proliferation. If prolonged, he says, that mechanism can spawn tumors in the bladder, lungs, and skin.

But Cohen insists this whole process depends on the generation of reactive arsenic metabolites that, in turn, interact with sulfhydryl groups in critical cell proteins. And at minimal doses (below 10 ppb in drinking water given to experimental animals or 100 ppb in well water consumed by humans, he says), arsenic exposure doesn’t generate enough reactive metabolites to induce tumor growth, suggesting that arsenic has a dose threshold. Moreover, Cohen claims that only direct reactions with DNA produce linear, nonthreshold dose responses for cancer, but according to the evidence, he says, inorganic arsenic is not DNA-reactive.^18^

“A linear dose–response line goes against what we know about arsenic’s basic biology,” Cohen says. “What we show in the lab shows there must be a threshold phenomenon.”

Other scientists disagree. Steinmaus, for instance, counters that rodents may not be good models for human arsenic metabolism given that “they don’t get cancer at doses that clearly cause cancer in humans.” He says, “You need to interpret those data cautiously.”

Moreover, high-dose human data from Taiwan are valuable because they remove some of the uncertainty associated with exposure, Steinmaus claims. Villagers in Taiwan often spend much of their lives in one general location, so the arsenic measured in local well water likely reflects their actual long-term intake. By contrast, populations in the United States and other developed countries with lower arsenic levels in groundwater are more mobile, leading to a strong likelihood of exposure misclassification. This statistical bias occurs when individual subjects in epidemiology studies are classified as having consumed more—or less—of a substance over a given duration than they actually ingested, making it difficult to accurately estimate disease associations.

Thus, the EPA SAB concluded in 2010 that—given the size and stability of the population, as well as the inclusion of long-term exposure patterns—the Taiwanese data were “still the most appropriate source for estimating bladder and lung cancer risk to humans.”^10^ But the SAB also stated that published studies from countries with low levels of arsenic in drinking water (which the SAB defined as up to 160 ppb) should be critically evaluated.^10^

## Evidence for Low-Dose Impacts

Low-dose studies are now ongoing in a number of countries, including various locations throughout the United States. For instance, in 2013 Ana Navas-Acien, an associate professor of environmental sciences and epidemiology at the Johns Hopkins Bloomberg School of Public Health, published results from a prospective study showing that urinary arsenic concentrations reflecting low and moderate drinking water exposures were associated with lung, prostate, and pancreatic cancer,^5^ as well as with cardiovascular disease,^2^ among Native Americans living in Arizona, Oklahoma, and the Dakotas.

Navas-Acien’s team measured arsenic in urine samples that had been collected and frozen between 1989 and 1991. The cohort of nearly 4,000 individuals had originally been assembled for the Strong Heart Study (SHS), an evaluation of cardiovascular health in Native Americans launched by the National Heart, Lung and Blood Institute in 1988. According to Navas-Acien, Native Americans included in the SHS tend to be more geographically stable than the general U.S. population, limiting the potential for exposure misclassification.^5^ “They get the same exposure to arsenic year after year that they got at birth,” she explains.

By matching local well water data collected by the EPA and urinary arsenic measures from the SHS samples with information from death certificates up through 2008, Navas-Acien could study the relationship between arsenic exposure and cancer mortality. Her team’s results suggested that arsenic had a linear dose response with lung cancer in particular, although Navas-Acien points out that confidence intervals were wide at doses below 5 ppb in well water, indicating uncertainty at the lowest exposure levels.

A similar linear response was also estimated for prostate and pancreatic cancer, but with even wider confidence intervals at the lowest doses. However, the excess relative risks estimated for prostate and pancreatic cancer in Navas-Acien’s study are much greater than they are for lung cancer; this is inconsistent with findings from other areas such as Taiwan, and therefore raises questions among some researchers about the validity of the findings. Navas-Acien’s team didn’t evaluate bladder cancer or skin cancer because of the small number of cases.

In a separate study of the same SHS cohort, Navas-Acien reported an association between low-dose arsenic exposures and higher rates of cardiovascular disease.^2^ That study is one of the first prospective cohort studies to evaluate arsenic-related cardiovascular risk, including both incidence and mortality, in a population from the United States.

These findings add to a wealth of data emerging from what could be the largest evaluation of arsenic toxicity yet undertaken: the Health Effects of Arsenic Longitudinal Study (HEALS), which launched in Bangladesh in 2000.^19^ Coordinated by Graziano and Habibul Ahsan, a professor of epidemiology, medicine, and human genetics at the University of Chicago, HEALS has over time assembled a cohort of tens of thousands of individuals living in the district of Araihazar, where arsenic levels measured in well water have ranged from nondetectible to more than 900 ppb.

The HEALS team first reported an association between arsenic and high blood pressure in 2007 at well water concentrations of 10–40 ppb. Since then, HEALS has yielded dozens of papers associating arsenic at levels below 50 ppb with health conditions including heart disease, hematuria (blood in the urine), and impaired lung function.^20^^,^^21^^,^^22^ Studies also showed that increased total urinary arsenic was associated with skin lesions such as melanosis and keratosis, which are known precursors to skin cancer.^23^^,^^24^ “HEALS is an ongoing effort, and we are expanding the design and questions that we can answer with longer follow-up,” Ahsan says.

## The Regulatory Landscape

Confronted with mounting evidence of arsenic’s low-dose effects, its commercial uses are being phased out. Of particular concern are uses in agriculture, which can result in potentially significant human exposures. According to a November 2013 NRC report, foods dominate human arsenic exposures when the levels in drinking water drop below 50 ppb (drinking water drives the exposures when its arsenic content exceeds that amount).^14^

In some cases, agricultural soils are naturally high in arsenic, but arsenical herbicides also can leave residues that accumulate in crops. Most of these herbicides have now been phased out (with some exceptions made for turfgrass and cotton),^25^^,^^26^ but what remains in soil from past applications has been especially problematic in apple orchards, where these herbicides were routinely used, and in rice grown on old cotton fields that were treated with the chemicals.

Arsenical feed additives used to promote growth and prevent disease in poultry and swine may also be problematic for human consumers. However, these additives are also being phased out. Production of the feed additive roxarsone ceased voluntarily in 2011 after the FDA detected inorganic arsenic in the livers of chickens that ate it.^27^ The manufacturers of roxarsone and two other arsenical feed additives have requested that the FDA withdraw approval of these products.^28^ The agency is currently considering a request to ban a fourth additive, nitarsone.^28^

Now the FDA is weighing how to impose standards for arsenic in foods. The proposed standard of 10 ppb in apple juice was a first step in this direction, but advocates with CU say the agency should go further by imposing a 120-ppb standard for inorganic arsenic in rice.^29^ In November 2012 CU published the results of a study showing that 223 samples of rice and rice-based products sold in the United States contained inorganic arsenic at concentrations ranging from 29.4 to 210 ppb.^30^ In addition, Dartmouth investigators reported that brown rice syrup, a sweetener, might expose consumers to “significant concentrations” of inorganic arsenic.^31^ (Arsenic tends to accumulate in the aleurone layer of the rice grain, which gives brown rice its color. This layer is removed to produce white rice.^31^)

The FDA followed up with its own report on 1,300 samples of rice and rice products, which found that concentrations of inorganic arsenic ranged from an average 0.1 µg per serving in infant formula to an average 7.2 µg per serving in brown rice.^32^ For perspective, Aaron Barchowsky, a professor of environmental and occupational health at the University of Pittsburgh School of Public Health, says that daily consumption of 3 liters of water at the 10-ppb standard amounts to a 30-µg dose of inorganic arsenic.

In a 6 September 2013 statement, the FDA said the amount of detectable arsenic in the sampled rice and rice products was too low to cause “any immediate or short-term adverse health effects.”^33^ FDA spokesperson Shelly Burgess says the statement referred to short-term effects only, and not skin, bladder, or lung cancer.

But Michael Crupain, director of *Consumer Reports*’ Food Safety and Sustainability Center, insists that chronic low-level exposures over time are still a concern, especially for infants fed formula made with brown rice syrup, which had the highest levels detected in CU’s survey. The FDA is now performing a draft risk assessment for arsenic in rice, which agency officials say could guide further actions.

Also on the regulatory front, the EPA is grappling with its numerical estimate of arsenic’s cancer potency. This value, known as a cancer slope factor (CSF), guides important regulatory policies, including clean-up standards at contaminated waste sites. Set in 1998, the original CSF for arsenic was based on nonmelanoma skin cancers observed in the early Taiwanese studies.

In 2010 the EPA proposed a revised CSF as part of an arsenic reassessment under the agency’s Integrated Risk Information System (IRIS).^10^ The proposed CSF, which was based on newer reports of associations with more dangerous lung and bladder cancers, was 17 times greater than the older value. But that new value was protested by industry and other affected stakeholders, even some of the agency’s own scientists. Griffin, of EPA Region 8, says that with the revised CSF, arsenic clean-up levels would drop 100-fold, which is below natural background levels of arsenic in western states.

The proposal was subsequently dropped by the EPA, and under a congressional mandate the agency is now revising its arsenic reassessment with a focus on both cancer and noncancer end points. On 7 November 2013 the NRC presented a report that the EPA will use for guidance in drafting a new IRIS document.^14^ Graziano, who chairs the NRC committee, says the EPA will submit the revised document by 2015. And at that point, he says, NRC scientists will review it to ensure that dose–response relationships between inorganic arsenic and its effects are “appropriately estimated and characterized.”

## Continued Debate

Meanwhile the debate over low-dose health risks from arsenic will likely continue on two fronts: how to apply mechanistic findings from animal and *in vitro* research to human responses, and how to address fundamental uncertainties in the human data.

A key question is whether the recent epidemiological literature supports estimates of cancer risk predicted from linear dose–response models. Dozens of studies over the last 15 years have investigated human cancer risk from arsenic exposure at sites around the world. According to a 2011 review published by Herman Gibb, president of environmental consulting firm Tetra Tech Sciences, these studies provide conflicting evidence, in part because the sample sizes needed to quantify risks at drinking water doses less than 100 ppb are larger than what’s ordinarily achievable.^34^

Steinmaus argues that the high-dose epidemiology data may ultimately be most suitable for risk assessment, “but when you extrapolate down from those doses, the risks are huge.” He adds, “This raises the question of whether linear extrapolations are suitable, and herein lies the big controversy.”

**Arsenic in U.S. Private Wells**

Despite the 10-ppb upper limit on inorganic arsenic in municipal water, neither the EPA nor state governments regulate arsenic in private wells. However, a 2001 EPA study found that 13 million U.S. residents get their drinking water from private wells that exceed the federal arsenic standard.^35^^,^^36^^,^^37^ Similarly, in 2009 the U.S. Geological Survey (USGS) tested 1,774 private wells nationwide and found that 6.8% of them exceeded the EPA standard (these are the most recent national data available on arsenic in private wells).^38^

According to Leslie DeSimone, a water quality specialist with the USGS, 90% of the exceedances were below 50 ppb, but measured concentrations ranged as high as 242 ppb, with the highest levels detected in the West, Midwest, parts of Texas, and New England. During a survey of Maine wells conducted in the mid to late 2000s, USGS scientists measured a concentration of 3,100 ppb in the coastal town of Danforth.^39^ Maine resides within a belt of arsenic-laden bedrock that extends north from New York.

Bill Wilber, chief of the USGS National Water Quality Assessment Program, says in some cases simply drilling a well introduces oxygen and other elements that alter the chemistry of the underlying geology, liberating arsenic from bedrock and allowing it to seep into the water column. “So you can find a big exceedance in one well and not in another that’s just fifty feet away,” Wilber says.

According to Andy Smith, state toxicologist with the Maine Division of Environmental Health, the cost of a home-based system to remove arsenic from well water ranges from $300 for a point-of-use system (i.e., at the faucet) to as much as $9,000 for point-of-entry systems that treat water for the whole household. Federal or state assistance to purchase these systems generally isn’t available, Smith says.
